# An Intensity-Demodulated Fiber-Optic Magnetometer Based on Nanostructured Magnetic Fluid-Filled Fluidic Photonic Crystal Fibers

**DOI:** 10.3390/nano14020221

**Published:** 2024-01-19

**Authors:** Liangquan Zhu, Huan Wang, Qijing Lin, Kun Yao, Dan Xian, Ping Yang, Na Zhao, Bian Tian, Zhuangde Jiang

**Affiliations:** 1 The State Key Laboratory for Manufacturing Systems Engineering, Xi’an Jiaotong University, Xi’an 710054, China; zhuliangquan@stu.xjtu.edu.cn (L.Z.);; 2 Xi’an Aerospace Composite Materials Institute, Xi’an 710025, China; 3 The Higher Educational Key Laboratory for Flexible Manufacturing Equipment Integration of Fujian Province, Xiamen Institute of Technology, Xiamen 361021, China; 4 Shandong Laboratory of Yantai Advanced Materials and Green Manufacturing, Yantai 265503, China; 5 Xi’an Jiaotong University (Yantai) Research Institute for Intelligent Sensing Technology and System, Xi’an Jiaotong University, Xi’an 710049, China

**Keywords:** fiber-optic magnetometer, photonic crystal fiber, magnetic fluid, fluidic sensor

## Abstract

An intensity-demodulated fiber-optic magnetometer is proposed and experimentally investigated, which is fabricated via fusion splicing a segment of photonic crystal fiber (PCF) between single-mode fibers (SMFs), with the cladding air holes of PCF filled with magnetic fluid. Using the magneto-optical properties of the magnetic fluid, the transmission spectrum is changed with an external magnetic field. Based on the intensity variations in the transmission spectrum, the magnetic field is detected, and a sensitivity of 0.238 dB/mT is obtained at 1550.03 nm with the length of PCF 5.5 cm. By converting light signals into electrical signals, a sensitivity of 0.003 V/mT is achieved. The fiber-optic magnetometer possesses the advantages of simple fabrication, compact/robust structure, and low cost.

## 1. Introduction

Magnetic field measurement has significant applications in various fields, ranging from industrial automation to medical diagnostics. Recently, fiber-optic magnetometers have garnered increased interest owing to the inherent advantages of compact size, distributed sensing capability, high sensitivity, and immunity to electromagnetic interference. Based on different magnetically sensitive materials, fiber-optic magnetometers can be categorized into three types, i.e., magnetostrictive materials, magneto-optical materials, and magnetic fluid materials [1]. Over the past decade, magnetic fluid-based fiber-optic magnetometers have gained considerable attention due to their high sensitivity, flexible integration capabilities, and low cost.

As a kind of stable colloidal system, Magnetic fluid comprises nanostructured particles coated with a surfactant suspended in a carrier liquid. When subjected to an external magnetic field, magnetic fluid exhibits diverse magneto-optical properties, including field-dependent transmission, tunable refractive index, the Faraday Effect, and birefringence [1,2,3]. Based on these unique properties, magnetic fluid has been widely used in optical filters [4], optical switches [5], and magnetic field sensing [6,7,8,9,10]. Combined with magnetic fluid, various fiber-optic magnetometers have been developed for magnetic field sensing. The schemes of these magnetometers include multimode fiber-optic interferometers [11], Fabry–Perot interferometers [12], Fiber grating [13], and Surface Plasmon Resonance [14].

While many promising results have been attained, most of these fiber-optic magnetometers are based on wavelength demodulation. For the sensor’s miniaturization and integration, intensity-modulated fiber-optic magnetometers are preferable. To realize intensity-demodulated magnetometers, Duan et al. [15] reported a fiber-optic magnetometer based on x zcS-tapered and multimode fiber, whose sensitivity is up to 1.130 dB/mT in 4–12 mT. Lu et al. [16] designed a fiber-optic magnetometer by using single serial-tilted-tapered fiber functionalized with magnetic fluid. The structure achieved stronger mode coupling with magnetic fluid and obtained a sensitivity of 0.336 dB/mT. Tao et al. [17] proposed a fiber-optic magnetometer in which SMF-No core Fiber (NCF)-Few mode fiber (FMF)-NCF structure integrated with magnetic fluid is used. The magnetometer has a sensitivity of 0.30782 dB mT in the range of 0–18 mT. However, all these magnetometers are fixed in the capillaries or other rigid containers that cannot change in shape, which limits their applications.

Constructed from an array of air holes embedded within solid materials, PCFs serve as an optimal platform for integrating liquid materials and supporting long-distance light–matter interactions. Based on PCF, various microfluidic photonic devices have been developed, like optical filters [18], modulators [19], and fiber-optic magnetometers [20,21,22]. However, for these fiber-optic magnetometers, optical spectrum analyzers (OSAs) are still needed to monitor optical power changes. OSAs have limited resolution and slow scanning rates, impacting demodulation resolution and speed in fiber-optic magnetometers.

In this work, an intensity-demodulated fiber-optic magnetometer based on the infiltration of magnetic fluid into PCF is proposed and experimentally demonstrated. PCFs offer a fluidic platform for integrating magnetic fluids, which enable long-distance light–matter interactions. Without an external encapsulation structure, this magnetometer is adaptable to various scenarios for precise detection. With the magneto-optical properties of magnetic fluids, the transmission spectrum is changing with the external magnetic field. By monitoring the intensity variation in the spectrum, a sensitivity of 0.238 dB/mT at 1550.03 nm is realized with the length of PCF 5.5 cm. By converting light signals into electrical signals, a sensitivity of 0.003 V/mT is obtained in the range of 0–8 mT. 

## 2. Materials and Methods

**Figure 1:**
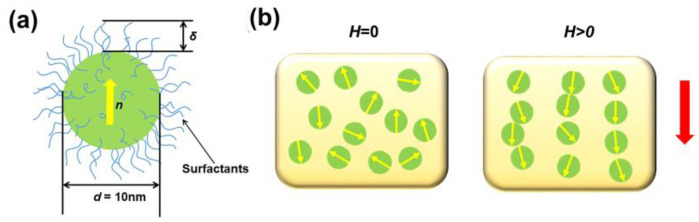
(**a**) Schematic of a ferromagnetic particle with surfactants; (**b**) schematic of the structural change in the magnetic fluid under an external magnetic field.

Magnetic fluids consist of ferromagnetic particles dispersed within a liquid carrier [23]. External magnetic fields can significantly influence the arrangement and properties of magnetic fluids, which is due to the interactions between the single-domain ferromagnetic particles within the magnetic fluid and the applied external magnetic field. These interactions result in significant changes in structural characteristics, consequently impacting the optical properties of the magnetic fluid. As depicted in Figure 1a, these ferromagnetic particles within magnetic fluid typically have a size of around 10 nm. In order to prevent the agglomeration of these particles caused by inter-particle interactions, the surface of which is coated with surfactants [23,24]. Surfactants are chemical substances that typically create a layer or coating around particle surfaces, reducing inter-particle attraction and consequently preventing aggregation or settling. This stabilization plays a significant role in achieving the homogeneous dispersion of ferromagnetic particles within a carrier liquid while retaining their inherent magnetic properties. As depicted in Figure 1b, the changes in the nanostructure of the magnetic fluid under the influence of an external magnetic field are demonstrated. In the absence of an external magnetic field, these ferromagnetic particles exhibit a disordered state. However, when exposed to an external magnetic field, these particles align themselves in the direction of the field, forming chain-like structures. Evidently, the rearrangement of these ferromagnetic particles significantly impacts their optical properties.

**Figure 2:**
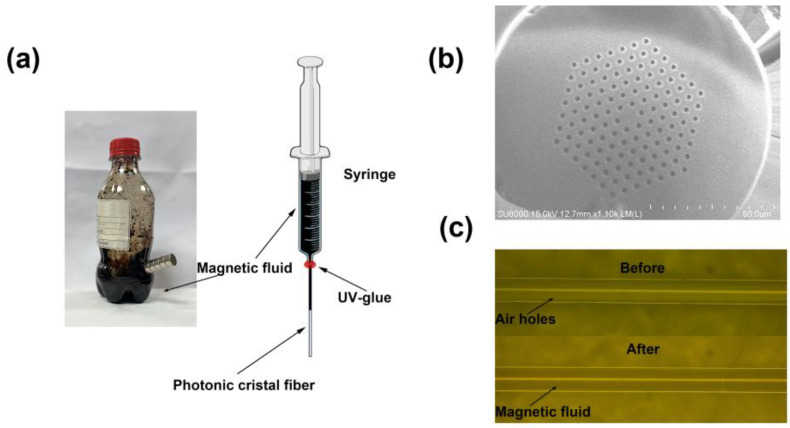
(**a**) Schematic of the process of filling magnetic fluid into PCF; (**b**) the cross section of the PCF; (**c**) the side view of PCF before and after being filled with magnetic fluid.

To fill magnetic fluid into the air holes of PCF, a short section of the fiber is initially prepared and sealed within a syringe using UV glue, as illustrated in Figure 2a. In the sealing process, it is crucial to avoid applying UV glue onto the end face of the fiber to prevent any unwanted adhesion. After pouring a small quantity of magnetic fluid into the syringe to submerge the fiber end, maintain a constant pressure to facilitate the filling process. After several hours, when the entire PCF changes color, it indicates the successful infusion of magnetic fluid into the PCF. The used magnetic fluid was provided by Hangzhou Jikang New Materials Co., Ltd. (Hangzhou, China) in the year of 2021, and contained ferromagnetic particles with a volume fraction of 0.33. A higher concentration of ferromagnetic particles has the potential to enhance sensor sensitivity [25]. However, when fusing SMF with PCF filled with a higher concentration of magnetic fluid, fusion deficiency occurred frequently at the fusion point, which significantly decreased the quality of fusion splicing and resulted in substantial light loss. Additionally, a higher concentration of ferromagnetic particles led to significant light absorption and scattering, causing large optical losses. Therefore, based on our experimental investigations, the used magnetic fluid was diluted by water to a ratio of about 1:9 (0.11). The cross section of the used PCF is shown in Figure 2b. And Figure 2c illustrates the side view of the photonic crystal fiber before and after the infusion of magnetic fluid.

**Figure 3:**
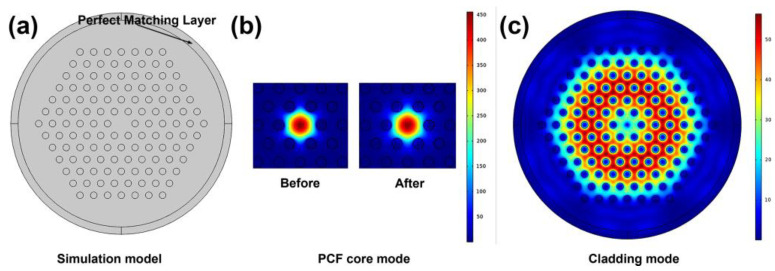
(**a**) The simulation model of PCF; (**b**) the simulation core modes of PCF before and after being filled with magnetic fluid; (**c**) the simulation cladding mode of the PCF after being filled with magnetic fluid.

To better understand the sensing mechanism of the fiber-optic magnetometer, the simulation of the fiber-optic modes has been conducted. The PCF model used in the simulation is illustrated in Figure 1a, employing perfectly matched layers as boundary conditions. After the magnetic fluid is filled, the effective refractive index of the cladding is increased. It can be seen from Figure 3b that the fiber core mode of PCF filled with magnetic fluid is not as concentrated as the PCF without magnetic fluid, leading to an expanded core mode area that enables the excitation of the cladding mode. In addition, as shown in Figure 3c, the cladding mode is also excited and demonstrates low-loss transmission characteristics. Consequently, the core mode and cladding mode may interfere.

## 3. Results and Discussion

**Figure 4:**
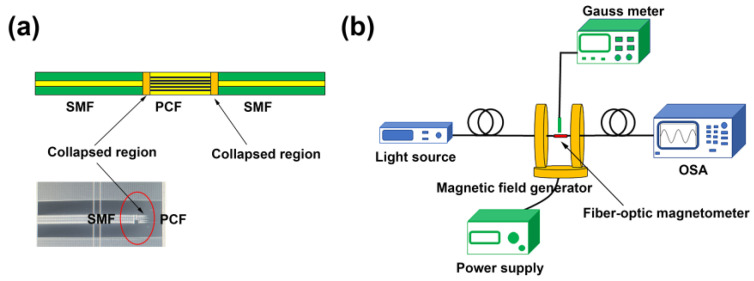
(**a**) Schematic of fiber-optic magnetometer structure; (**b**) experimental setup.

Figure 4a illustrates the structure of the fiber-optic magnetometer, comprising a short section of PCF filled with magnetic fluid and two SMFs. The fusion splicing of the PCF with SMFs was accomplished using a FITEL S178A fusion splicer in manual mode. The discharge electrical intensity and discharge time were set at 100 and 300, respectively. Before the fusion splicing, a mild arc discharge (100 ms) was generated several times using the fusion splicer to clean the fiber ends and to evaporate part of the magnetic fluid in the PCF. The fusion point of the SMFs and the PCF was shifted approximately 75 compensation distances away from the midpoint of the arc discharge point. That can avoid the occurrence of bubbles in the fusion point, which impacts the fusion quality. When subjected to the high temperature of the fusion splicing, the micro holes of PCF tend to collapse. Due to the collapse of micro holes in PCF, a collapsed region would form at the fusion joint. At this fusion point, part of the light would enter the clad of the PCF, causing interference with the light transmitted in the PCF core in the second SMF [11]:

(1) $$I=I_{core}+I_{clad}+2\sqrt{I_{core}I_{clad}}\cos\left(\frac{2\pi L}{\lambda }\times \Delta n_{eff}\right)$$

where *I_core_* and *I_clad_* denote the intensities of the PCF core mode and cladding modes; *L* represents the length of the PCF, while the Δ*n_eff_* signifies the effective refractive index difference between PCF core (*n_core_*) and cladding (*n_clad_*). The transmission spectrum dip wavelength can be expressed as

(2) $$\lambda_{dip}=\frac{2\Delta n_{eff}\cdot L}{2m+1}$$

(3) $$\Delta n_{eff}=n_{core}-n_{clad}$$

In the experiment, as shown in Figure 4b, two Helmholtz coils (CHY12-500, CH-Magnetoelectricity Technology Company, Beijing, China) were used as the magnetic field generator. The magnetic field generator can produce a uniform magnetic field with a sphere area of about 100 mm in diameter. The intensity of the magnetic field was manually adjusted using the DC power supply and calibrated using a Gauss meter. Light from the light source passed through the fiber-optic magnetometer and was recorded by the spectrometer. The used OSA provided by Anritsu Corporation (MS9740A, Tokyo, Japan), with a resolution of 0.03 nm. In the measurement, the Sample point is set at 1001, and the frequency is set as 100 Hz. 

**Figure 5:**
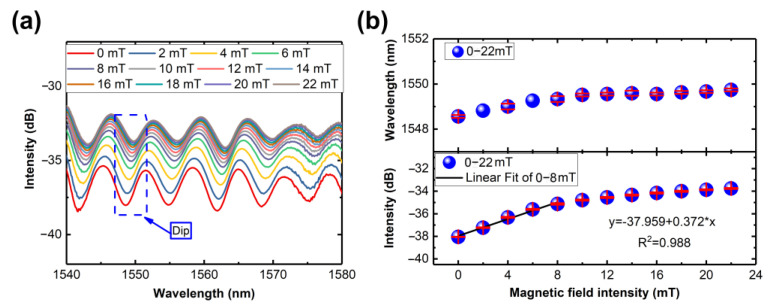

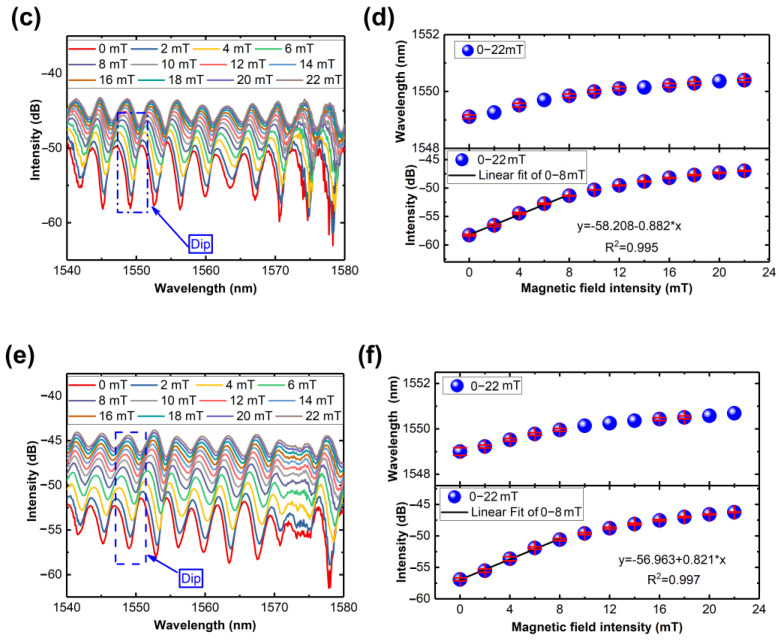
(**a**) The response of 5.5 cm PCF at different magnetic field intensities. (**b**) The linear fitting of the dip intensities at magnetic field intensities. (**c**) The response of 9.5 cm PCF at different magnetic field intensities. (**d**) The linear fitting of the dip intensities at magnetic field intensities. (**e**) The response of 9.5 cm PCF at different magnetic field intensities. (**f**) The linear fitting of the dip intensities at magnetic field intensities.

To assess the performance of the fiber-optic magnetometer, three sensors were fabricated using PCFs with lengths of about 5.5 cm, 9.5 cm, and 9.5 cm, respectively. In the measurement, the magnetometer was positioned at the center of the magnetic field generator, aligned parallel to the external magnetic field, as depicted in Figure 4b. The magnetic field was incrementally raised from 0 mT to 22 mT in intervals of 2 mT. At each measurement point, the transmission spectrum was recorded by OSA and analyzed using the computer. The corresponding response at different magnetic field intensities is illustrated in Figure 5a,c,d. With the magnetic field intensity increasing, the intensity of the transmission spectrum dips gradually increased while the wavelength slightly moved to a longer wavelength. The reduction in spectral transmission loss is due to the magnetic field altering the optical properties of the magnetic fluid [26], i.e., the changes in absorption, scattering coefficients, refractive index, etc. The light intensity can also be written as follows:

(4) $$I=I_{PCF}\exp\left(-\alpha L\right)$$

where α represents the extinction coefficient directly associated with the absorption and scattering coefficients of the magnetic fluid. And *I_PCF_* represents the total transmitted light in the PCF.

The magnetic field can induce changes in the extinction coefficient of magnetic fluid, thereby causing variations in the transmission losses within PCFs. In addition, with the refractive index of magnetic fluid within the PCF changes, the effective refractive index difference between PCF core and cladding changes, and thus Δ*n_eff_* shifts *δn* accordingly, leading to the displacement of the transmission spectrum dip by an estimated amount *δλ*.

(5) $$\delta \lambda \approx \frac{2L\delta n}{2m+1}$$

Figure 5b,d,f show the intensity changes and wavelength shifts of transmission spectrum dip. In the measurements, every experiment was repeated five times. During the data processing phase, three repetitions were used for data processing. It can be obtained that in the ranges of 0–8 mT, the magnetometers have magnetic field sensitivity of 0.372 dB/mT, 0.882 dB/mT, and 0.821 dB/mT, respectively, while the wavelength changes are less than 1 nm. And the corresponding maximum standard errors are 0.12702, 0.21027, and 0.31442. The variance in sensitivity between two sensors of the same length might arise from discrepancies resulting from manual splicing fusion. Due to the fusion splicing machine being unable to identify and fuse PCFs, the fusion splicing of PCF and SMF operated in manual mode. Manual fusion splicing requires precise alignment, and due to possible machine errors and the small core size (around 9 μm) of the fibers, achieving complete uniformity among sensors becomes challenging. And various studies have reported that intentional misalignment during fusion splicing will change the sensitivity [11,25,26,27].

**Figure 6:**
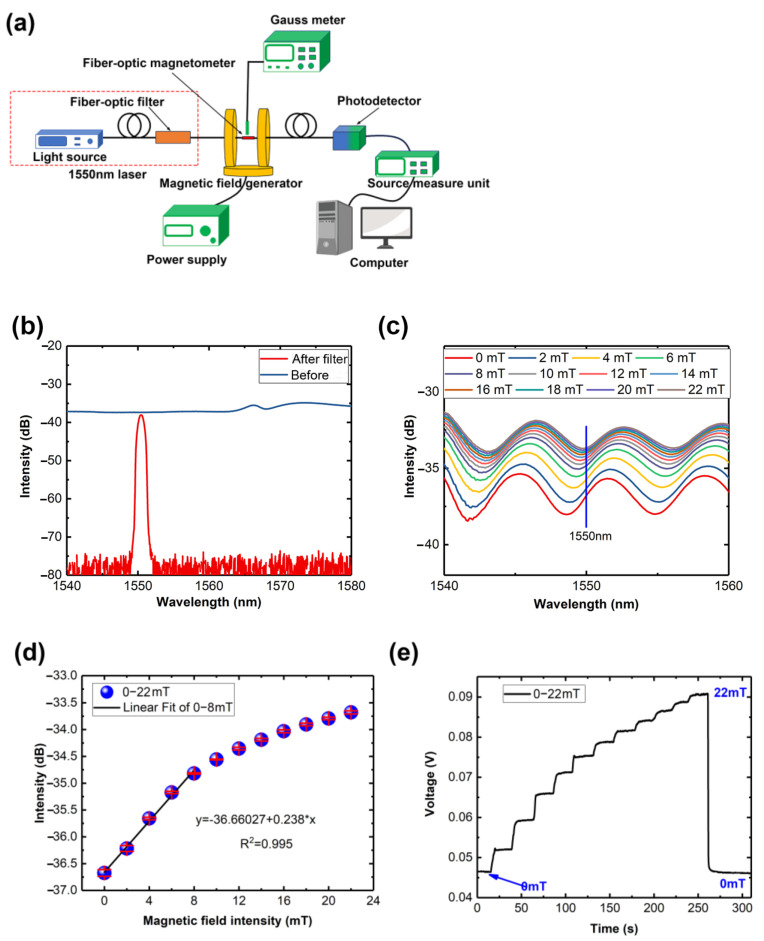

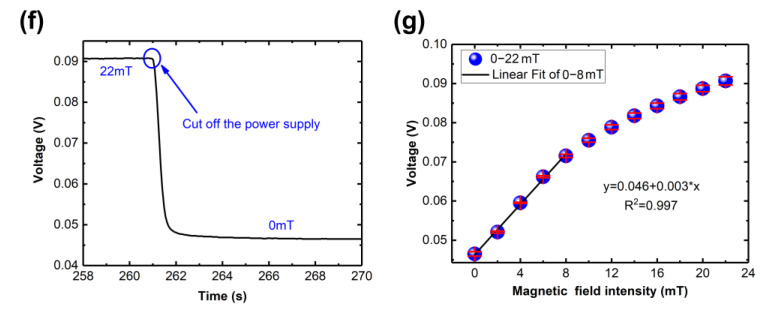
(**a**) Experimental setup; (**b**) the spectrum of the light source after passing through a fiber-optic filter; (**c**) the spectrum of 5.5 cm PCF at different magnetic field intensities near 1550 nm; (**d**) linear fitting of intensities of 1550.3 nm at different magnetic field intensities. (**e**) The process of increasing the magnetic field from 0 to 22 mT; (**f**) the recovery time of fiber-optic magnetometer from 22 mT to 0 mT; (**g**) linear fitting of the output voltages at different magnetic field intensities.

Intensity change signals are easier converted into electrical signals, thus reducing sensor costs and enabling sensor miniaturization. As shown in Figure 6a, a combination of a fiber-optic filter (HE1550-C, Beijing H&E Information Tech Co., Ltd., Beijing, China), photodetector (KG-30KHz-A-FC, Beijing Conquer Photonics Co., Ltd., Beijing, China), and source measure unit were utilized to convert light signals into electrical ones. The fiber-optic filter selectively allows light near 1550 nm to pass through, filtering out other wavelengths. As depicted in Figure 6b, the light source emits light after passing through the fiber-optic filter, mainly consisting of 1550 nm wavelengths. 

As shown in Figure 5a,c,e, the spectrum intensity of the 5.5 cm PCF fiber-optic magnetometer consistently increases near 1550 nm. Then, data near 1550 nm of the 5.5 cm PCF fiber-optic magnetometer were extracted from Figure 5a and depicted in Figure 6c, revealing a sensitivity of 0.238 dB/mT (Figure 6d) with a maximum error of 0.06156 in the region of 0–8 mT at 1550.03 nm. Similar to the previous experiment, the magnetic field intensity gradually increased up to 22 mT. The entire process is depicted in Figure 6e. The output voltage is increasing corresponding to the external magnetic field, which corresponds to the increased light intensity near the wavelength of 1550 nm. Additionally, Figure 6f illustrates the recovery time span from 22 mT to 0 mT, which lasts approximately 1 s for the entire span. Within the magnetic field range of 0–8 mT, as shown in Figure 6g, the fiber-optic magnetometer exhibits a sensitivity of 0.003 V/mT, with a maximum standard error of 5.56776 × 10^−4^. The reason that the slope of electrical sensitivity is not consistent with the light intensity signal at 1550.03 nm may be due to the broader bandwidth of the fiber-optic filter. 

The performance comparison of our fiber-optic magnetometer with others is listed in Table 1. Due to the intensity demodulation used, it can be more easily converted into electrical signals compared to other magnetometers based on wavelength demodulation [27,28], thus facilitating cost reduction and miniaturization. In contrast to other intensity-demodulated fiber-optic magnetometers, the proposed fiber-optic magnetometer still presents a good sensitivity in a single wavelength of 1550.03 nm, and the conversion of light signals into electrical signals is realized. 

**Figure 7:**
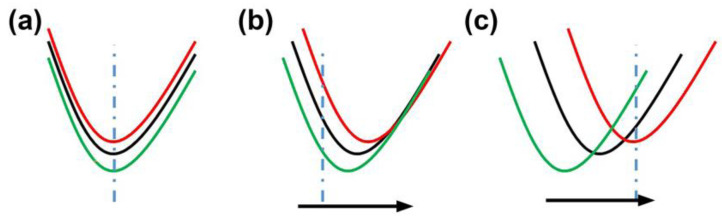
Schematic of the spectrum: (**a**) unchanged; (**b**) minor shifts; (**c**) substantial shifts.

Furthermore, it is worth noting that the transmission spectrum dip’s wavelength exhibits insensitivity to alterations in the magnetic field. The observed wavelength shift of the dip remains small, staying within a range of only 1 nm over the 0–8 mT range. As depicted in Figure 7, when the spectrum wavelength remains consistent, the choice of the detection point for light-intensity demodulation becomes flexible. Any wavelength can be chosen as the detection point. When dealing with minor shifts, there still exists a broad range of wavelengths that can be selected as the detection point. However, in cases of substantial shifts, the available region of wavelengths suitable for selection diminishes considerably. Thus, the intensity-demodulated fiber-optic magnetometers with small wavelength shifts enhance the feasibility of achieving a high-sensitivity fiber-optic magnetometer.

The manual control of the fusion splicing method in this article makes it difficult to ensure high repeatability. Therefore, for the fabrication of fiber-optic magnetometers with greater consistency, future studies should consider adopting more precise fusion splicing methods. It is worth noting that the ferromagnetic particles within the magnetic fluid tend to form chain-like structures aligned with the direction of the magnetic field, leading the fluid to demonstrate anisotropic properties when influenced by an external magnetic field [10]. As the direction of the magnetic field changes, the chains within the magnetic fluid rotate correspondingly, resulting in alterations of optical characteristics in magnetic fluid. When the sensor is aligned with the magnetic field, the spectrum intensity increases, indicating a decrease in the extinction coefficient within the PCF. When the sensor is not aligned with the magnetic field, the inherent anisotropic properties of magnetic fluid may cause an increase in the extinction coefficient, thus decreasing the spectral intensity. Effectively utilizing the anisotropy of magnetic fluids will contribute to creating vector fiber-optic magnetometers. Additionally, a systematic study of the light-matt interaction within PCFs will contribute to the production of high-sensitivity fiber-optic magnetometers.

## 4. Conclusions

A fiber-optic magnetometer based on the tunable magneto-optical properties of the magnetic fluid is demonstrated. The magnetic fluid-filled PCF proved to be a reliable medium for magnetic field sensing, capitalizing on the magneto-optical properties of the magnetic fluid. By monitoring the intensity of the transmission spectrum, magnetic field information is obtained. A sensitivity of 0.238 dB/mT is attained in a single wavelength of 1550.03 nm. The conversion of light signals into electrical signals was carried out, and a sensitivity of 0.003 V/mT was realized. The proposed fiber-optic magnetometer offers simple fabrication, compact/robust structure, and low cost. 

## Figures and Tables

**Table 1:** The performance comparison of fiber-optic magnetometer.

Structure	Linear Region	Sensitivity	Photoelectric Conversion	Refs
SMF-PCF-SMF (2018)	2–20 mT	0.13 dB/mT (spectrum dip)	No	[20]
SMF-PCF-SMF (2020)	0–10 mT	0.92463 nm/mT (spectrum dip)	No	[27]
SMF-PCF-SMF (2020)	0–140 mT	0.021 dB/mT (spectrum dip)	No	[22]
SMF-PCF-SMF (2023)	2–30 mT	0.37dB/mT (spectrum dip)	No	[21]
SMF-PCF-Reflective end (2023)	6–15 mT	1.16 nm/mT (spectrum dip)	No	[28]
Our structure	0–8 mT	0.238 dB/mT (1550.03 nm)	0.003 V/mT	This work

## Data Availability

The original contributions presented in the study are included in the article. Further inquiries can be directed to the corresponding author.
